# Initial and Recurrent Hyperkalemia Events in Patients With CKD in Older Adults: A Population-Based Cohort Study

**DOI:** 10.1177/20543581211017408

**Published:** 2021-05-27

**Authors:** Sriram Sriperumbuduri, Eric McArthur, Gregory L. Hundemer, Mark Canney, Navdeep Tangri, Silvia J. Leon, Sara Bota, Ann Bugeja, Ayub Akbari, Greg Knoll, Manish M. Sood

**Affiliations:** 1 Department of Medicine, University of Ottawa, ON, Canada; 2 Ottawa Hospital Research Institute, ON, Canada; 3 International Council for the Exploration of the Sea, Ottawa, ON, Canada; 4 Chronic Disease Innovation Centre, Seven Oaks General Hospital, Winnipeg, MB, Canada; 5 Department of Internal Medicine, Max Rady College of Medicine, University of Manitoba. Winnipeg, Canada

**Keywords:** hyperkalemia, eGFR, albuminuria, chronic kidney disease, epidemiology, RAAS

## Abstract

**Background:**

The risk of hyperkalemia is elevated in chronic kidney disease (CKD); however, the initial and recurrent risk among older individuals is less clear.

**Objectives:**

We set out to examine the initial and 1-year recurrent risk of hyperkalemia by level of kidney function (estimated glomerular filtration rate, eGFR) in older adults (≥66 years old).

**Design:**

Population-based, retrospective cohort study

**Settings:**

Ontario, Canada

**Participants:**

905 167 individuals (≥66 years old) from 2008 to 2015.

**Measurements:**

Serum potassium values

**Methods:**

Individuals were stratified by eGFR (≥90, 60-89, 30-59, 15-29 mL/min/1.73 m^2^) and examined for the risk of incident hyperkalemia (K ≥ 5.5 mEq/L) using adjusted Cox proportional hazards models. The 1-year risk of recurrent hyperkalemia was examined using multivariable Andersen-Gill models.

**Results:**

Among a population of 905 167 individuals (15% eGFR ≥ 90, 58% eGFR 60-89, 25% eGFR 30-59, 3% eGFR 15-29) with a potassium measurement, there were a total of 18 979 (2.1%) individuals with hyperkalemia identified. The event rate (per 1000 person-years) and adjusted hazard ratio (HR) of hyperkalemia was inversely associated with eGFR (mL/min; eGFR >90 mL/min: 8.8, referent, 60-89 mL/min: 11.8 HR 1.41; eGFR 30-59: 39.8, HR 4.37; eGFR 15-29: 133.6, 13.65) and with an increasing urine albumin-to-creatinine ratio (ACR, mg/mmol; ACR< 3: 14, referent, ACR 3-30: 35.1, HR 1.98; ACR >30: 93.7, 4.71). The 1-year event rate and adjusted risk of recurrent hyperkalemia was similarly inversely associated with eGFR (eGFR ≥ 90: 10.1, referent, eGFR 60-89: 14.4, HR 1.47; eGFR 30-59: 54.8, HR 4.90; eGFR 15-29: 208.0, HR 12.98). Among individuals with a baseline eGFR of 30 to 59 and 15 to 29, 0.9 and 3.8% had greater than 2 hyperkalemia events. The relative risk of initial and recurrent hyperkalemia was marginally higher with RAAS blockade. Roughly 1 in 4 individuals with hyperkalemia required hospitalization the day of or within 30 days after their hyperkalemia event.

**Limitations:**

Limited to individuals aged 66 years and above.

**Conclusions:**

Patients with low eGFR are at a high risk of initial and recurrent hyperkalemia.

**Trial registration:**

**N/A**

## Abrégé

**Contexte:**

Le risque d’hyperkaliémie est élevé en contexte d’insuffisance rénale chronique (IRC). On en sait cependant peu sur le risque initial et récurrent d’hyperkaliémie chez les patients âgés.

**Objectif:**

Nous avons examiné le risque initial d’hyperkaliémie et le risque de récurrence sur une année selon le niveau de fonction rénale (débit de filtration glomérulaire estimé [DFGe]) chez les patients âgés (plus de 66 ans).

**Type d’étude:**

Étude de cohorte rétrospective basée sur une population.

**Sujets:**

L’étude porte sur un total de 905 167 individus (âgés de 66 ans et plus) entre 2008 et 2015.

**Mesures:**

Les valeurs de potassium sérique.

**Méthodologie:**

Les individus ont été stratifiés en fonction du DFGe (≥90, 60-89, 30-59, 15-29 ml/min/1.73m^2^) et examinés pour le risque d’hyperkaliémie incidente (K ≥ 5,5 mEq/L) à l’aide de modèles de risques proportionnels de Cox corrigés. Le risque de récurrence sur un an a été examiné avec des modèles multivariés d’Andersen-Gill.

**Résultats:**

Parmi les 905 167 individus disposant d’une mesure de potassium sérique (15 % avec un DFGe ≥ 90; 58 % avec un DFGe de 60-89; 25 % avec un DFGe de 30-59; et 3 % avec un DFGe de 15-29), on a recensé 18 979 individus (2,1 %) présentant une hyperkaliémie. Le taux d’événements (pour 1 000 années-personnes) et le rapport de risque corrigé (RR) de l’hyperkaliémie étaient inversement associés au DFGe (ml/min). Ainsi, un DFGe > 90 ml/min a été associé à 8,8 événements pour 1 000 années-personnes (EV) et constituait le référent pour le RR; ces valeurs pour les autres niveaux de DFGe étaient les suivantes: 11,8 EV (RR = 1,41) pour un DFGe 60-89; 39,8 EV (RR = 4,37) pour un DFGe de 30-59; et 133,6 EV (RR = 13,65) pour un DFGe de 15-29. L’accroissement de ces valeurs a également été associé à un accroissement du rapport albumine/créatinine dans l’urine (RAC mg/mmol). Ainsi, un RAC < 3 a été associé à 14 EV et constituait le référent pour le RR, tandis que 35,1 EV (RR = 1,98) ont été observés pour un RAC de 3-30; et que ce nombre passait à 93,7 EV (RR=4,71) pour un RAC > 30. Le taux d’événements sur un an et le risque corrigé d’hyperkaliémie récidivante étaient eux aussi inversement associés au DFGe: 10,1 EV (RR = valeur de référence) pour un DFGe ≥ 90; 14,4 EV (RR=1,47) pour un DFGe 60-89; 54,8 EV (RR=4,90) pour un DFGe 30-59; et 208,0 EV (RR=12,98) pour un DFGe 15-29. Parmi les individus présentant un DFGe initial de 30-59 et de 15-29 ml/min/1,73m^2^, un certain nombre de patients avaient vécu plus de deux événements d’hyperkaliémie (respectivement 0,9 % et 3,8 %). Le risque relatif d’hyperkaliémie initiale et récurrente était légèrement plus élevé avec le blocage du SRAA. Environ une personne sur quatre atteinte d’hyperkaliémie a dû être hospitalisée le jour de l’événement ou dans les 30 jours suivant celui-ci.

**Limites:**

L’étude a été limitée aux personnes âgées de 66 ans et plus.

**Conclusion:**

Les patients présentant un faible taux de DFGe présentent un risque élevé d’hyperkaliémie initiale et récurrente.

## What was known before

Hyperkalemia is a life threatening and often recurrent event requiring medical management as many of the causes of hyperkalemia are incurable, chronic conditions.

## What this adds

Examining a large population of older individuals, the risk of incident and recurrent hyperkalemia was higher with declining eGFR. In individuals with an eGFR 15 to 29 mL/min/1.73 m^2^ the risk for incident and 1-year recurrent hyperkalemia were roughly 13-fold higher compared to individuals with intact kidney function. One-quarter of individuals with hyperkalemia required hospitalization within 30 days.

## Background

Hyperkalemia is one of the most commonly encountered electrolyte disorders with prevalence estimates in the general population of 1.6% to 6.6% and is associated with an elevated risk of mortality and hospitalization.^1-3^ Identified risk factors include diabetes mellitus, congestive heart failure (CHF), and use of renin angiotensin aldosterone system inhibitor (RAASi) medications,^4,5^

Chronic kidney disease, defined by a reduction in eGFR for greater than 90 days or persistent albuminuria, is a well-identified risk factor for hyperkalemia.^1-10^ As many of the risk factors for hyperkalemia are irreversible, there is a persistent risk of recurrence.^11-13^ This is of particular concern among older adults, in whom the prevalence of both CKD and risk factors for hyperkalemia is highest,^14,15^ Older adults appear to have a confluence of hyperkalemia risk factors (heart failure, diabetes, medications) and physiologic processes associated with aging such as reduced renal potassium handling with reduced renal mass, impaired potassium secretion from the distal nephron, and a higher prevalence of hyporeninemic hypoaldosteronism that increase their susceptibility.^16-18^ The burden of hyperkalemia and its risk of recurrence is less well characterized at the population level. This is important as hyperkalemia may be a harbinger for adverse outcomes, can lead to discontinuation of medications with proven benefit such as angiotensin converting enzyme (ACE) inhibitors or angiotensinogen receptor blockers (ARB) and contributes to increased health care resources and cost.^2,4,19,20^

We sought to define the incidence of hyperkalemia as a function of eGFR in older adults in the general population and estimate the risk of recurrence of hyperkalemia by eGFR level.

## Methods

### Setting

We conducted a population-level, retrospective cohort study of adults over the age of 65 years from July 1, 2008 to August 31, 2015 in Ontario, Canada. Ontario is Canada’s largest province with over 14.5 million residents.^21^ All citizens have access to universal public health care with drug coverage for individuals over the age of 65 years. This study was conducted using a pre-specified protocol and reporting of the results and adheres to the Reporting of Studies Conducted Using Observational Routinely-Collected Health Data (RECORD) guidelines (Supplemental Table 1).^22^ The use of de-identified data in this project was authorized under section 45 of Ontario’s Personal Health Information Protection Act, which does not require review by a Research Ethics Board.

### Data Sources

We ascertained patient characteristics, medication data, and outcome data from de-identified linked databases housed at ICES.^23^ Demographics and vital status information were obtained from the Ontario Registered Persons Database. Medication information was obtained from the Ontario Drug Benefit database.^24^ Diagnostic and procedural information from all hospitalizations was determined using the Canadian Institute for Health Information Discharge Abstract Database (CIHI-DAD). Diagnostic information from emergency room visits was determined using the CIHI National Ambulatory Care Reporting System (CIHI-NACRS). Laboratory data were obtained from the Ontario Laboratories Information System which captures laboratory measures from hospital and community laboratories in Ontario.^25^ Additional information was also obtained from the Ontario Health Insurance Plan database, which contains all health claims for inpatient and outpatient physician services. Whenever possible, we defined patient characteristics and outcomes using validated codes.

### Cohort Definition

All adults ≥66 years of age with an outpatient serum creatinine and a urine albumin-to-creatinine (ACR) measurement were included (for study cohort creation see Supplemental Figure 1, for exposure and outcome definitions see Supplemental table 2). The first eligible outpatient serum creatinine measurement served as the study index date while the urine ACR measure preceding by up to 365 days. Serum creatinine was converted to eGFR using the Chronic Kidney Disease Epidemiology Collaboration (CKD-EPI) equation with both eGFR and urine ACR reported as categories using the Kidney Disease: Improving Global Outcomes (KDIGO) guidelines.^26^ Prescription drug information is available for all adults ≥65 years of age in Ontario, and we initiated our cohort at age 66 years to allow for a 1-year look back period for pre-existing medications. We excluded individuals with a history of hyperkalemia in the preceding 6 months (to capture incident events), those receiving renal replacement therapy (RRT, defined as dialysis or kidney transplantation) or kidney failure (eGFR <15 mL/min/1.73 m^2^) without RRT.

### Exposure

The primary study exposure was kidney function defined by the KDIGO categories of eGFR (≥90, 60-89, 30-59, 15-29 mL/min/1.73 m^2^).^27^ Kidney function was defined by the first eligible single outpatient eGFR measure.^28^ We further examined albuminuria categorized as urine ACR categories (<3, 3-30, >30 mg/mmol) for the initial hyperkalemia episode. Patients were censored at study outcome (first and recurrent hyperkalemia events), death, or maximum follow-up date (1 year follow-up from index or the end of data availability [September 30, 2017]).

### Covariates

Potential confounders examined included demographics (age, sex, neighborhood income), index year, co-morbid illnesses (hypertension, diabetes, stroke, acute coronary syndrome, heart failure, coronary artery disease, coronary artery bypass grafting, peripheral vascular disease), and medications (ACE or ARB, beta-blocker, Nonsteroidal anti-inflammatory drugs [NSAID], potassium-sparing diuretics, aldosterone receptor antagonists, low-molecular-weight heparin, non-potassium-sparing diuretics, and statins).

### Outcomes

Our primary outcome was a first measure of hyperkalemia defined as serum potassium ≥5.5 mEq/L. Secondary outcomes were recurrent episodes of hyperkalemia based on measures up to 1 year of follow-up and risk factors for the initial episode of hyperkalemia. We further examined the proportion of individuals who were hospitalized (all-cause) at the time of or shortly after a hyperkalemia event and the median length of stay by eGFR category. One year was chosen as it could represent a reasonable follow-up time for an interventional trial. Potassium measures within 24 hr of the index date were excluded and values occurring within 2 days of each other were considered part of the same episode.

### Statistical Analysis

Baseline characteristics were examined stratified by eGFR exposure categories (≥90, 60-89, 30-59, 15-29 mL/min/1.73 m^2^). Continuous variables are reported as means with standard deviations or median values with 25th to 75th percentile interquartile range and categorical variables as frequencies (percentages). We calculated the incidence rate (defined as the rate per 1000 person-years of follow-up) for the outcomes of interest. We examined the association of eGFR and ACR categories (separately) and a first episode of hyperkalemia using Cox proportional hazards models treating mortality as a censoring event. Models were adjusted for potential confounders listed above. To examine the risk of recurrent hyperkalemia, we used the Andersen-Gill (AG) model adjusted for the same variables as above for the total cohort. The AG model allows those who experienced an event to continue to contribute time at risk (and subsequent events) following their first event.^29^ We further stratified our recurrent events model by the use of an angiotensin converting enzyme (ACE) inhibitor or angiotensin II receptor blocker (ARB). We conducted all analyses with SAS software, version 9.4 (SAS Institute Inc., Cary, NC, USA). Two-sided *P* values <.05 were considered statistically significant.

## Results

### Baseline Characteristics

A total of 9 076 230 people from Ontario, Canada had a serum creatinine test in OLIS between 2008 and 2015. Those with age <66 years, eGFR <15 mL/min/1.73 m^2^, an episode of hyperkalemia 6 months prior to the day of recruitment and renal transplant recipients were excluded from the study. The final study population included 905 167 patients with an outpatient serum creatinine level and urine ACR within 12 months of index creatinine. The majority of individuals had an eGFR 60 to 89 mL/min/1.73 m^2^ (58%) followed by eGFR 30 to 59 mL/min/1.73 m^2^ (25%), eGFR ≥ 90 (15%) and eGFR 15 to 29 mL/min/1.73 m^2^ (3%) (Table 1). Women comprised 53% of the cohort and the mean age was 74 years. Subjects with an eGFR 15 to 29 mL/min/1.73 m^2^ were older with mean age of 79 ± 8 years and 71% of these subjects were older than 75 years of age. In the entire population, 55% had history of diabetes, 78% had hypertension, 41% had coronary artery disease, and 3% had stroke within 5 years prior to the index date. Coronary artery disease and stroke were more frequent with lower eGFR categories. Regarding medications, 63% of the subjects were on an ACE/ARB, 3% were on potassium sparing diuretic, and 11% were prescribed NSAIDs. Baseline use of ACE/ARB and spironolactone was higher in subjects with worse renal dysfunction at 70% and 10%, respectively, with eGFR <60 mL/min/1.73 m^2^ vs. 56% and 1%, respectively, with eGFR ≥60 mL/min/1.73m^2^. Low eGFR was also associated with increased albuminuria (urine ACR > 30 mg/mmol) with a prevalence of 26% in subjects with eGFR 15 to 29 mL/min/1.73m^2^ compared to 2% in subjects with eGFR ≥ 90 mL/min/1.73 m^2^.

**Table 1.** Baseline Characteristics of the Study Cohort by Estimated Glomerular Filtration Rate Level.

	Estimated glomerular filtration rate (mL/min/1.73 m^2^)			
*Characteristics*	**≥90**	**60 to 89**	**30 to 59**	**15 to 29**
Total	133 697	521 472	222 844	27 154
*Demographics*				
Age				
Mean ± SD	68 ± 3	73 ± 6	77 ± 7	79 ± 8
Median (IQR)	67 (66–69)	72 (68–77)	77 (71–82)	80 (73–85)
65 to <75	125 714 (94%)	329 409 (63%)	88 512 (40%)	7814 (29%)
75 to <85	7742 (6%)	162 685 (31%)	96 685 (43%)	11 812 (44%)
≥85	241 (0%)	29 378 (6%)	37 647 (17%)	7528 (28%)
Sex, N (%)				
Female	69 404 (52%)	256 507 (49%)	120 797 (54%)	15 096 (56%)
Income quintile, N (%)				
Quintile 1—lowest	24 889 (19%)	96 064 (18%)	46 138 (21%)	6037 (22%)
Quintile 2	27 683 (21%)	107 664 (21%)	48 409 (22%)	6018 (22%)
Quintile 3	26 985 (20%)	104 749 (20%)	44 427 (20%)	5392 (20%)
Quintile 4	27 975 (21%)	107 966 (21%)	43 859 (20%)	5147 (19%)
Quintile 5—highest	25 675 (19%)	103 525 (20%)	39 319 (18%)	4448 (16%)
Missing	490 (0%)	1504 (0%)	692 (0%)	112 (0%)
Residential Status, N (%)				
Rural	15 384 (12%)	59 561 (11%)	25 938 (12%)	3338 (12%)
Missing	<6 (0.0%)	<6 (0.0%)	8 (0.0%)	<6 (0.0%)
Year of index date, N (%)				
2008	3715 (3%)	15 471 (3%)	7963 (4%)	1355 (5%)
2009	12 961 (10%)	72 012 (14%)	36 483 (16 %)	4777 (18%)
2010	22 757 (17%)	115 146 (22%)	55 793 (25%)	7038 (26%)
2011	20 292 (15%)	84 636 (16%)	35 629 (16%)	4148 (15%)
2012	20 553 (15%)	72 829 (14%)	28 363 (13%)	3395 (13%)
2013	21 479 (16%)	69 981 (13%)	25 795 (12%)	2765 (10%)
2014	20 206 (15%)	59 212 (11%)	21 278 (10%)	2368 (9%)
2015	11 734 (9%)	32 185 (6%)	11 540 (5%)	1308 (5%)
Long-term care resident, N (%)	806 (1%)	4102 (1%)	3231 (1%)	828 (3%)
**Comorbidities, 5 years prior to index date**				
Albumin-to-creatinine ratio (mg/mmol), N (%)				
<3	109 840 (82%)	420 759 (81%)	145 281 (65%)	9940 (37%)
3–30	20 969 (16%)	87 762 (17%)	60 950 (27%)	10 230 (38%)
>30	2888 (2%)	12 951 (3%)	16 613 (8%)	6984 (26%)
Coronary artery disease with angina	32 301 (24%)	156 202 (30%)	89 115 (40%)	13 673 (50%)
Coronary artery bypass grafting	1716 (1%)	8910 (2%)	5316 (2%)	736 (3%)
Congestive heart failure	6090 (5%)	38 063 (7%)	36 797 (17%)	8944 (33%)
Myocardial infarction	2502 (2%)	13 395 (3%)	10 370 (5%)	2346 (9%)
Stroke & transient ischemic attack	1665 (1%)	9274 (2%)	6728 (3%)	1254 (5%)
Atrial fibrillation/flutter	2852 (2%)	20 100 (4%)	17 600 (8%)	3622 (13%)
Peripheral vascular disease	987 (1%)	4761 (1%)	4568 (2%)	1019 (4%)
Venous thromboembolism	34 649 (26%)	143 162 (28%)	80 140 (36%)	12 655 (47%)
Hypertension	90 470 (68%)	384 612 (74%)	189 199 (84%)	23 907 (88%)
Diabetes	78 014 (58%)	265 301 (51%)	121 409 (55%)	15 439 (57%)
Aldosterone and renin disorders	42 (0%)	206 (0%)	150 (0%)	31 (0%)
Chronic liver disease	6241 (5%)	18 222 (4%)	7837 (4%)	1107 (4%)
Cancer	4194 (3%)	15 232 (3%)	7199 (3%)	941 (4%)
No. of hospitalizations				
Median (IQR)	0 (0–0)	0 (0–0)	0 (0–0)	0 (0–1)
No. of emergency department visits				
Median (IQR)	0 (0–0)	0 (0–1)	0 (0–1)	0 (0–1)
No. of nephrology visits				
Median (IQR)	0 (0–0)	0 (0–0)	0 (0–0)	1 (0–2)
**Medications, 120 days prior to index date, N (%)**				
Nonsteroidal anti-inflammatory drugs	20 100 (15%)	80 782 (16%)	38 678 (17%)	3754 (14%)
ACE or ARB	72 354 (54%)	296 179 (57%)	157 022(71%)	19 010 (70%)
Beta blockers	25 928 (19%)	130 477 (25%)	83 334 (37%)	13 358 (49%)
Statins	70 228 (53%)	281 765 (54%)	135 111(61%)	17 585 (65%)
Potassium-sparing diuretics	1047 (1%)	5994 (1%)	7679 (3%)	1909 (7%)
Mineralocorticoid receptor antagonists	943 (1%)	5522 (1%)	7328 (3%)	1857 (7%)
Low molecular weight heparin	352 (0. %)	1195 (0%)	727 (0 %)	139 (1%)
Non-potassium sparing diuretics	23 619 (18%)	116 487 (22%)	87 460 (39%)	16 911 (62%)

*Note.* IQR = interquartile range; mg = milligram; mmol = millimole; ACE = angiotensin converting enzyme inhibitors; ARB = angiotensin-receptor blockers.

### Association of eGFR, Albuminuria, and First Hyperkalemia Event

**Figure 1.**
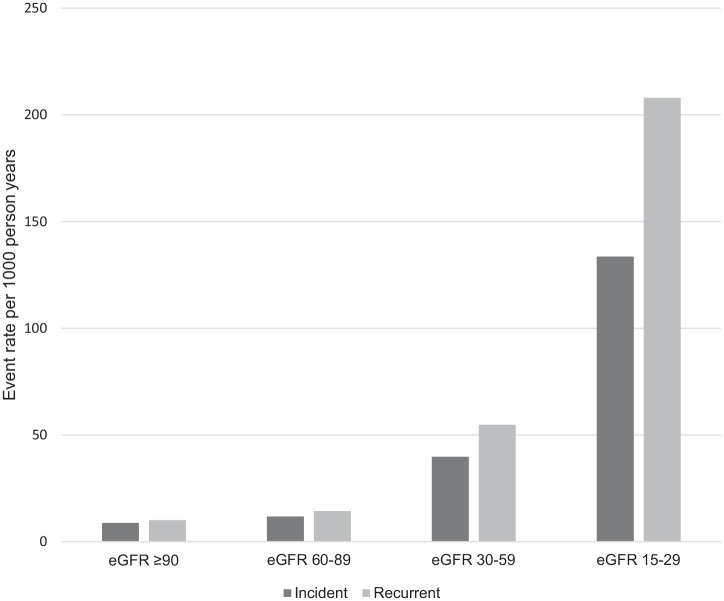
Crude incident and recurrent event rates (per 1000 person-years) of hyperkalemia (≥5.5 mEq/L) by CKD categories. *Note.* CKD = chronic kidney disease; eGFR = estimated glomerular filtration rate.

A total of 18 979 individuals (2.1%) had an incident episode of hyperkalemia (see Table 2) with a stepwise increase in hyperkalemia with lower eGFR and higher ACR. The 1-year population attributable risk of a first hyperkalemia event was 59% for individuals with an eGFR 89 mL/min/1.73 m^2^ or lower compared to individuals with an eGFR ≥ 90 mL/min/1.73 m^2^. The crude percentage for initial hyperkalemia within 1 year was 0.9%, 1.2%, 3.8%, and 11.8% for eGFR categories of ≥90, 60 to 89, 30 to 59, and 15 to 29 mL/min/1.73 m^2^, respectively (corresponding crude rates per 1000 person-years were 8.8, 11.8, 39.8, 133.6, see Figure 1). The adjusted hazard ratio (HR) demonstrated a similar graded increase (eGFR ≥ 90 mL/min/1.73 m^2^: HR referent, 60-89 mL/min/1.73 m^2^: HR 1.41 [95% CI = 1.32-1.50], 30-59 mL/min/1.73 m^2^: HR 4.37 [95% CI = 4.10-4.66] and 15-29 mL/min/1.73 m^2^: HR 13.65 [95% CI = 12.69-14.68]). Hyperkalemia was increased with higher ACR categories (ACR mg/mmol <3; 1.4%, ACR 3-30 mg/mmol: 3.4%, ACR >30 mg/mmol: 8.7%) and this persisted in adjusted models (ACR <3 mg/mmol; HR referent ACR 3-30 mg/mmol: HR 1.98 [95% CI = 1.92-2.05], ACR >30 mg/mmol: HR 4.71 [95% CI = 4.52-4.90]).

**Table 2.** The Number of Events, Event Rate, and Hazard Ratio of an Initial Hyperkalemia Event by Estimated Glomerular Filtration Rate Level.

Estimated glomerular filtration rate	N	Number of events (%)	Event rate per 1000 person-years	Unadjusted hazard ratio (95% confidence interval)	Adjusted^a^ hazard ratio (95% confidence interval)
**≥**90 mL/min/1.73 m^2^	133 697	1157 (0.9%)	8.8	Reference	
60 to 89 mL/min/1.73 m^2^	521 472	6072 (1.2%)	11.8	1.35 (1.27 to 1.44)	1.41 (1.32 to 1.50)
30 to 59 mL/min/1.73 m^2^	222 844	8534 (3.8%)	39.8	4.54 (4.27 to 4.83)	4.37 (4.10 to 4.66)
15 to 29 mL/min/1.73 m^2^	27 154	3216 (11.8%)	133.6	15.28 (14.28 to 16.34)	13.65 (12.69 to 14.68)

a Model adjusted for age (per year), sex (male referent), income quintile (highest quintile referent), cerebrovascular disease (stroke/transient ischemic attack), myocardial infarction, coronary artery disease, coronary artery bypass grafting, hypertension, congestive heart failure, diabetes, chronic obstructive pulmonary disease, peripheral vascular disease, year of index date (2007 referent), and all baseline medications.

### Association of eGFR and Recurrent Hyperkalemia Events

Of the 18 979 individuals with an initial hyperkalemia event, 22% (4096) had a recurrent hyperkalemia event over the 1-year follow-up. With decreasing eGFR categories, a stepwise increase in the risk of recurrent hyperkalemia events was noted. Among individuals with intact eGFR (eGFR >60 mL/min/1.73 m^2^), the risk of 1 or more recurrent hyperkalemia event(s) was 0.1% compared to 0.9% and 3.8% in those with an eGFR of 30 to 59 and <30 mL/min/1.73 m^2^, respectively; 5.3% of those with eGFR 15 to 29 mL/min with a hyperkalemia event had 3 or more subsequent episodes within the year. The corresponding crude rates per 1000 person-years were eGFR > 90 mL/min 10.1, eGFR 60 to 89 mL/min 14.4, eGFR 30 to 59 mL/min 54.8 and eGFR 15 to 29 mL/min 208 (see Figure 1). The adjusted risk of hyperkalemia was exceedingly high among those with an eGFR of 15 to 29 mL/min/1.73 m^2^ (HR 12.98, [95% CI = 12.06-13.96]) with an attenuated but elevated risk remaining among whose with eGFR 30 to 59 mL/min/1.73 m^2^ (HR 4.90 [95% CI = 4.61-5.20]) and eGFR 60-89 mL/min/1.73 m^2^ (HR 1.47 [95% CI = 1.39-1.56], eGFR ≥ 90 mL/min/1.73 m^2^ referent, see Table 3). The risk of hyperkalemia differed by use of an ACE/ARB (+ACE/ARB: eGFR of 15-29 mL/min/1.73 m^2^ HR 13.51 [95% CI = 12.47-14.64], eGFR 30-59 mL/min/1.73 m^2^ HR 5.26 [95% CI = 4.89-5.66], eGFR = 60-90 mL/min/1.73 m^2^ HR 1.57 [95% CI = 1.46-1.70], eGFR ≥ 90 mL/min/1.73 m^2^ referent; ACE/ARB-: eGFR 15-29 mL/min/1.73 m^2^ HR 11.75 [95% CI = 10.45-13.21], eGFR 30-59 mL/min/1.73 m^2^ HR 4.06 [95% CI = 3.67-4.50], eGFR 60-90 mL/min/1.73 m^2^ HR 1.30 [95% CI = 1.18-1.43], eGFR ≥ 90 mL/min/1.73 m^2^ referent, interaction *P* < .0001).

**Table 3.** The Number of Events, Event Rate, and Hazard Ratio of a Recurrent Hyperkalemia Event by Estimated Glomerular Filtration Rate Level.

Estimated glomerular filtration rate	N	Total number of events	Event rate per 1000 person-years	Unadjusted hazard ratio (95% confidence interval)	Adjusted^a^ hazard ratio (95% confidence interval)
**≥**90 mL/min/1.73 m^2^	133 697	1338	10.1	Reference	
60 to 89 mL/min/1.73 m^2^	521 472	7410	14.4	1.45 (1.37 to 1.54)	1.47 (1.39 to 1.56)
30 to 59 mL/min/1.73 m^2^	222 844	11 762	54.8	5.49 (5.19 to 5.81)	4.90 (4.61 to 5.20)
15 to 29 mL/min/1.73 m^2^	27 154	5007	208.0	19.88 (18.71 to 21.13)	12.98 (12.06 to 13.96)

a Model adjusted for age (per year), sex (male referent), income quintile (highest quintile referent), cerebrovascular disease (stroke/transient ischemic attack), myocardial infarction, coronary artery disease, coronary artery bypass grafting, hypertension, congestive heart failure, diabetes, chronic obstructive pulmonary disease, peripheral vascular disease, year of index date (2007 referent), and all baseline medications.

### Risk Factors Associated With an Initial Hyperkalemia Event

**Figure 2.**
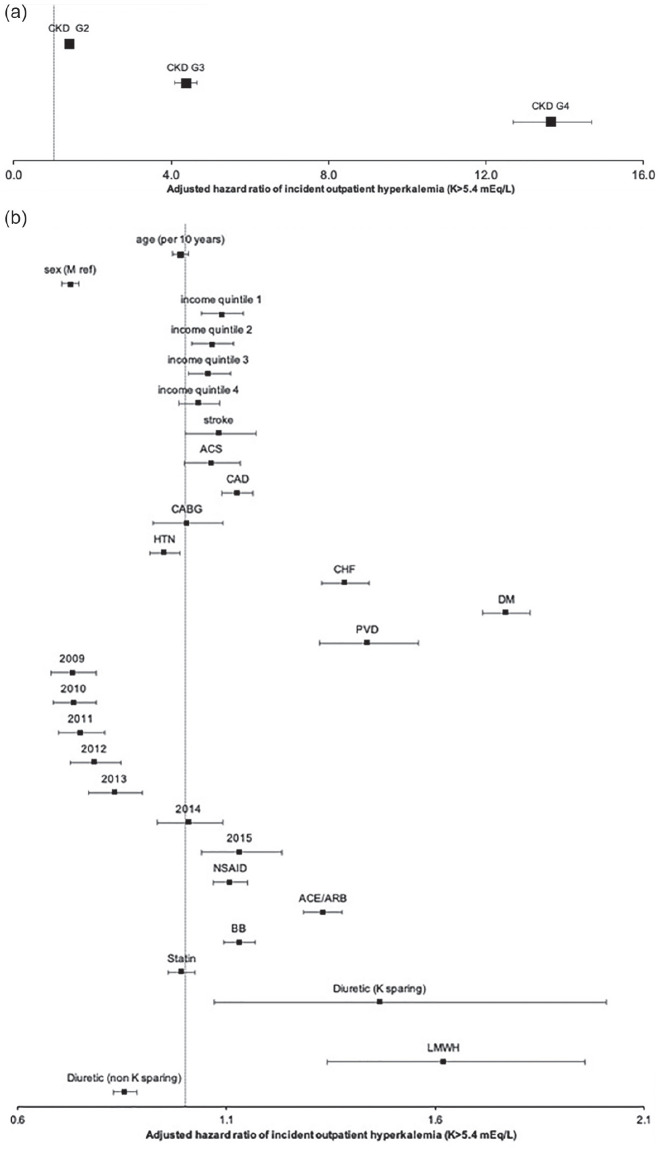
Forest plot of variables associated with an incident hyperkalemia event: (a) association of CKD categories with hyperkalemia (CKD G1 referent) and (b) association of other factors with hyperkalemia. *Note.* Model was adjusted for age (per year), sex (male referent), income quintile (highest quintile referent), cerebrovascular disease (stroke/transient ischemic attack), myocardial infarction, coronary artery disease, coronary artery bypass grafting, hypertension, congestive heart failure, diabetes, peripheral vascular disease, year of index date (2007 referent), and medications (all baseline meds). Referent: CKD category eGFR **≥** 90, Sex male, Income quintile highest quintile, index year 2008. CKD = chronic kidney disease; Q = quintile; CABG = coronary artery bypass grafting; HTN = hypertension; CHF = congestive heart failure; PVD = peripheral vascular disease; NSAID = non-steroidal anti-inflammatory; RAASi = renin angiotensin aldosterone inhibitor defined by ACE inhibitor or angiotensinogen receptor blocker use; K = potassium; LMWH = low molecular weight heparin; eGFR = estimated glomerular filtration rate; ACE = angiotensin converting enzyme inhibitors.

A number of risk factors were associated with a higher risk of an initial hyperkalemia event among individuals with different eGFR levels (see Figure 2). CKD G4 (eGFR of 15-29 mL/min/1.73 m^2^) was the single largest risk factor (HR 13.65 95% CI 12.69-14.68, referent eGFR ≥ 90 mL/min/1.73 m^2^), followed by eGFR 30-59 mL/min/1.73 m^2^, diabetes mellitus, low-molecular-weight heparin, potassium-sparing diuretics, eGFR 60-89 mL/min/1.73 m^2^, congestive heart failure, ACE/ARB use, coronary artery disease, beta-blocker use, lower income, stroke, and myocardial infarction. Women, hypertension, and non-potassium sparing diuretic use were associated with a lower risk of hyperkalemia.

### All-Cause Hospitalization and Median Length of Stay After an Outpatient Hyperkalemia Event

Among all hyperkalemia events, 19.1% were inpatient events with the highest crude proportion in those with an eGFR ≥ 90 mL/min/1.73 m^2^ (27.0%) and comparable proportions in those with lower eGFR categories (eGFR 60-89 mL/min/1.73 m^2^: 18.6%, eGFR 30-59 mL/min/1.73 m^2^: 17.9%, eGFR 15 to 29 mL/min/1.73 m^2^: 20.5%). The median length of hospital stay was 11 (IQR 4-28), 9 (IQR 4-21), 9 (IQR 4-22), and 12 (IQR 5-30) days for eGFR groups of ≥90, 60 to 89, 30 to 59, and 15 to 29 mL/min/1.73 m^2^, respectively. When we examined hospitalizations among individuals with more severe hyperkalemia (K > 6.5 mEq/L), the findings were consistent (eGFR group, median length of stay: ≥ 90: 5.6%, 6.5 [IQR 4-20], 60-89: 4.9%, 6 [3-16.5], 30-59: 4.8%, 7[3-19], and 15-29: 4.4%, 7[3-18] days).

## Discussion

In this retrospective cohort of 905 167 older individuals, we observed a graded increase in 1-year risk for hyperkalemia events (first and recurrent) by declining eGFR categories. The elevated risk was consistent after adjusting for a large number of potential confounders and was associated with a number of pertinent risk factors. Among individuals with CKD G4 (eGFR 15 to 29 mL/min/1.73 m^2^), the 1-year risk of initial and recurrent hyperkalemia was exceedingly high with over a 13-fold higher risk compared to those with intact kidney function and this risk further exacerbated by ACE or ARB use. Hyperkalemia had important clinical consequences as 1 in 4 individuals with an initial hyperkalemia event had at least 1 recurrent event within the year and 1 in 4 hyperkalemia events were associated with hospitalization.

The current study is consistent with and expands on our previous understanding of the epidemiology of outpatient hyperkalemia,^5,8,13^ Chang et al examined hyperkalemia (using the same definition as the current study of >5.5 mEq/L) in 194 456 outpatients in a large U.S. health care system reporting a frequency of 2.3% over a 3-year period (comparable with the 2.1% over 1 year we observed in both in-and outpatients).^8^ Kidney function was similarly inversely associated with hyperkalemia and the strongest predictor of its occurrence. Furthermore, they examined the duration of hyperkalemia defined as transient (based on 1 measure), intermittent (2 or 3 measures per year), and persistent (4 or more measures per year) with 3-year proportions in individuals with an eGFR < 30 mL/min/1.73m^2^ of 12%, 19%, and 30%, respectively. Adelborg et al examined 262 375 individuals for hyperkalemia (defined as >5 mEq/L) in a high-risk population including both in- and outpatient measures in Denmark.^13^ An exceedingly high 37% to 49% had a recurrent event within 6 months depending on incident RAASi use, a history of CKD or CHF. Using the SCREAM registry in Stockholm, Sweden Nilsson et al reported an occurrence of 2.5% of K >5.5 in 364 955 individuals with the strongest predictor being an eGFR of <30 mL/min/1.73 m^2^ (adjusted odds ratio 6.39 [95% CI = 5.93-6.89]).^30^ A meta-analysis of 27 international cohorts including 10 CKD cohorts (CKD prognosis consortium), reported a prevalence of hyperkalemia of 0.49% 95% CI = 0.48 to 0.50 in the general population/high cardiovascular (CV)-risk cohort and 4.23% 95% CI = 4.03 to 4.42 in the CKD cohort.^6^ Direct comparisons between studies are difficult based on the varying study designs, the mix of in- and outpatient measurements used, disparate definitions of hyperkalemia, and length of follow-up. We specifically examined a K > 5.5 as it is more strongly associated with clinically relevant outcomes and linked to actionable changes in clinical care.^6^ Furthermore, we limited our follow-up time to 1 year as it represents a plausible time frame for a clinical intervention to reduce hyperkalemia recurrence.

We identified a number of hyperkalemia-associated risk factors including CKD categories, diabetes, CHF, cardiovascular disease, and the use of medications that inhibit/reduce renal potassium excretion. These risk factors are remarkably consistent across studies examining different cohorts; however, the magnitude of the association with lower eGFR groups in our cohort was high (eGFR 30-59 mL/min/1.73 m^2^: HR 4.37, eGFR <30 mL/min/1.73 m^2^: HR 13.65).^11-13,31^ This may be a reflection of our focus on individuals over 66 years of age (mean cohort age 74 years) with roughly 8% of our study population being ≥85 years of age. This is in contrast to most other studies reporting the epidemiology of hyperkalemia which included individuals over a wider age range (≥18 years).

Nearly 1 in 4 individuals with a hyperkalemia episode required hospitalization with a median hospital length of stay of between 9 and 12 days depending on eGFR. This highlights the common role of hyperkalemia in multiple acute illnesses and its role as a marker of illness severity. Our findings were consistent with Adelborg et al where 30% of the patients with one hyperkalemic event required hospitalization.^13^ Horne et al reported a strong independent association with hyperkalemia and all-cause hospitalization in the primary care setting in England (HR 28.93 95% CI = 27.22-30.72).^9^

The current study has a number of limitations. We did not examine drug doses for implicated medications or whether changes in prescriptions occurred after a primary hyperkalemia event. As such, we are unable to comment on the role of medication or therapeutic changes associated with recurrent hyperkalemia episodes. We are unable to differentiate “true” hyperkalemia events from false or pseudo hyperkalemia events. We did not examine changes in kidney function over time and it is plausible that a number of events occurred after changes in baseline kidney function (either acutely or chronically). We did not include information on confounders such as race, insulin use, urine output, frailty, oral potassium binders, or dietary potassium intake.^32,33^ We were unable to account for over-the-counter availability of NSAIDs. Despite the large number of covariates accounted for in our analysis, residual confounding may persist, and we present associations that may not be causal. Our cohort inclusion criteria of requiring an ACR measure or age greater than 66 may limit generalizability. Patients with lower eGFR values may be more closely monitored and thereby hyperkalemia more likely to be detected (indication bias). Finally, we used single measures of outpatient eGFR and ACR to define our baseline classifications which are subject to potential misclassification (AKI as CKD).

In conclusion, we report the risk of a primary and/or recurrent hyperkalemia events in a population of advanced age with different levels of kidney function. Advanced CKD (eGFR 15-29 mL/min/1.73m^2^), diabetes, cardiovascular disease, and medications that alter potassium handling were significantly associated with hyperkalemia events. Our findings provide reliable estimates of incidence and risk in older people by leveraging our large dataset with multiple linkages thereby allowing informed discussions about the potential risks and benefits in older individuals with a diminishing eGFR.

## Supplemental Material

sj-pdf-1-cjk-10.1177_20543581211017408 – Supplemental material for Initial and Recurrent Hyperkalemia Events in Patients With CKD in Older Adults: A Population-Based Cohort Study Click here for additional data file. Supplemental material, sj-pdf-1-cjk-10.1177_20543581211017408 for Initial and Recurrent Hyperkalemia Events in Patients With CKD in Older Adults: A Population-Based Cohort Study by Sriram Sriperumbuduri, Eric McArthur, Gregory L. Hundemer, Mark Canney, Navdeep Tangri, Silvia J. Leon, Sara Bota, Ann Bugeja, Ayub Akbari, Greg Knoll and Manish M. Sood in Canadian Journal of Kidney Health and Disease
