# Supplementary material for: Initial and Recurrent Hyperkalemia Events in Patients With CKD in Older Adults: A Population-Based Cohort Study
Source: Can J Kidney Health Dis. 2021 May 27;8:20543581211017408. doi: 10.1177/20543581211017408 (PMC8165829; doi:10.1177/20543581211017408)
Supplement: sj-pdf-1-cjk-10.1177_20543581211017408 – Supplemental material for Initial and Recurrent Hyperkalemia Events in Patients With CKD in Older Adults: A Population-Based Cohort Study [file sj-pdf-1-cjk-10.1177_20543581211017408.pdf]

Supplementary Figures & Tables

Supplementary Figure 1. Cohort creation flow diagram

Supplementary Table 1: REporting of studies Conducted using Observational Routinely-collected health Data (RECORD) statement checklist

Supplementary Table 2. Key administrative data definitions

Supplemental Table 3. Risk factors for first hyperkalemia event

Supplementary Figures & Tables

Supplementary Figure 1. Cohort creation flow diagram

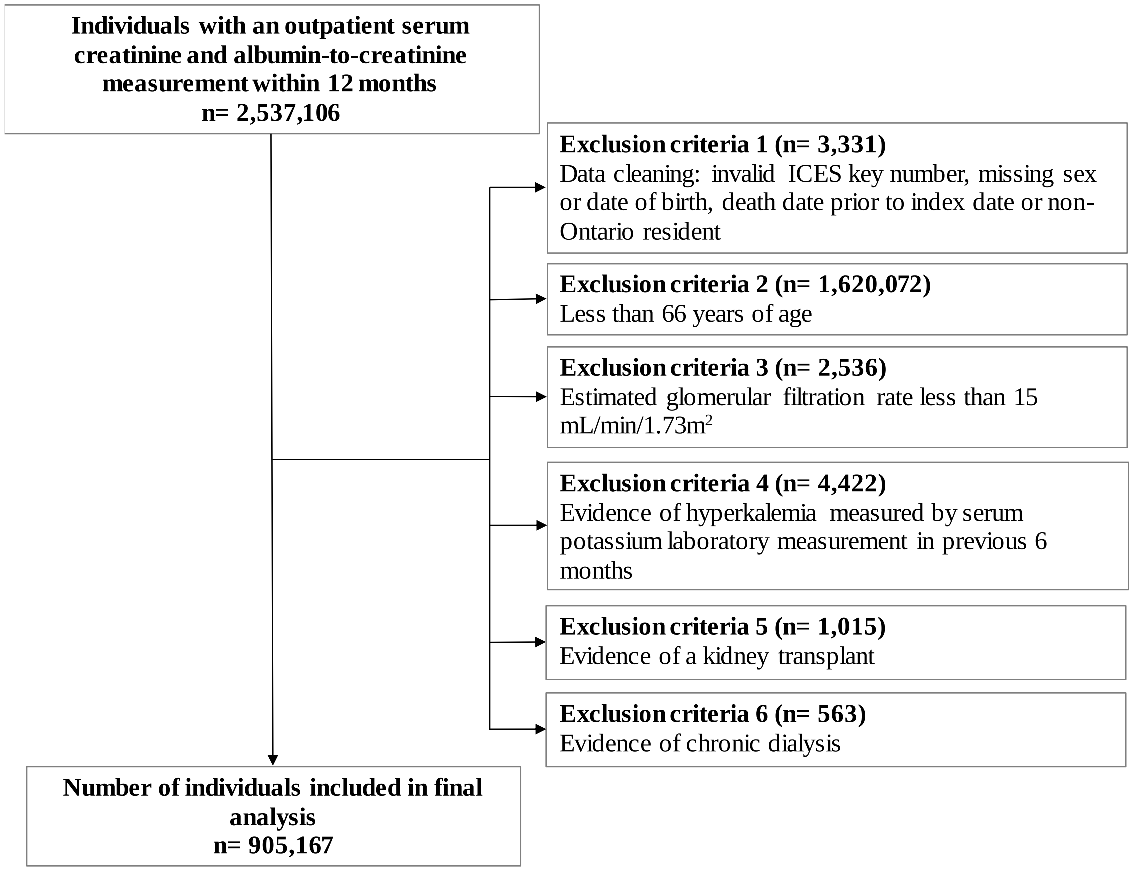

Supplementary Table 1: Reporting of studies Conducted using Observational Routinely-collected health Data (RECORD) statement checklist

	Item No	STROBE items	RECORD items	Reported
**Title and abstract**	1	(a) Indicate the study's design with a commonly used term in the title or the abstract.<br>(b) Provide in the abstract an informative and balanced summary of what was done and what was found.	(1.1) The type of data used should be specified in the title or abstract. When possible, the name of the databases used should be included.<br>(1.2) If applicable, the geographic region and time frame within which the study took place should be reported in the title or abstract.<br>(1.3) If linkage between databases was conducted for the study, this should be clearly stated in the title or abstract.	Title page
**Introduction**				
Background/ rationale	2	Explain the scientific background and rationale for the investigation being reported.		Introduction
Objectives	3	State specific objectives, including any prespecified hypotheses.		Introduction
**Methods**				
Study design	4	Present key elements of study design early in the paper.		Methods
Setting	5	Describe the setting, locations, and relevant dates, including periods of recruitment, exposure, follow-up, and data collection.		Methods
Participants	6	(a) Give the eligibility criteria, and the sources and methods of selection of participants. Describe methods of follow-up.<br>(b) For matched studies, give matching criteria and number of exposed and unexposed.	(6.1) The methods of study population selection (such as codes or algorithms used to identify subjects) should be listed in detail. If this is not possible, an explanation should be provided.<br>(6.2) Any validation studies of the codes or algorithms used to select the population should be referenced. If validation was conducted for this study and not published elsewhere, detailed methods and results should be provided.<br>(6.3) If the study involved linkage of databases, consider use of a flow diagram or other graphical display to demonstrate the data linkage process,	Methods

			including the number of individuals with linked data at each stage.	
Variables	7	Clearly define all outcomes, exposures, predictors, potential confounders, and effect modifiers. Give diagnostic criteria, if applicable.	(7.1) A complete list of codes and algorithms used to classify exposures, outcomes, confounders, and effect modifiers should be provided. If these cannot be reported, an explanation should be provided.	Methods
Data sources/ measurement	8	For each variable of interest, give sources of data and details of methods of assessment (measurement). Describe comparability of assessment methods if there is more than one group.		Methods
Bias	9	Describe any efforts to address potential sources of bias.		Methods
Study size	10	Explain how the study size was arrived at.		Methods/eFigure 1
Quantitative variables	11	Explain how quantitative variables were handled in the analyses. If applicable, describe which groupings were chosen and why.		Methods
Statistical methods	12	(a) Describe all statistical methods, including those used to control for confounding.<br>(b) Describe any methods used to examine subgroups and interactions.<br>(c) Explain how missing data were addressed.<br>(d) If applicable, explain how loss to follow-up was addressed.<br>(e) Describe any sensitivity analyses.		Methods
Data access and cleaning methods		N/A	(12.1) Authors should describe the extent to which the investigators had access to the database population used to create the study population.<br>(12.2) Authors should provide information on the data cleaning methods used in the study.	Methods
Linkage		N/A	(12.3) State whether the study included person-level, institutional-level, or other data linkage across two or more databases. The methods of linkage and methods of linkage quality	Methods

			evaluation should be provided.	
**Results**				
Participants	13	(a) Report numbers of individuals at each stage of study--e.g. numbers potentially eligible, examined for eligibility, confirmed eligible, included in the study, completing follow-up, and analyzed.<br>(b) Give reasons for non-participation at each stage.<br>(c) Consider use of a flow diagram.	(13.1) Describe in detail the selection of the persons included in the study (i.e., study population selection), including filtering based on data quality, data availability, and linkage. The selection of included persons can be described in the text and/or by means of the study flow diagram.	Results
Descriptive data	14	(a) Give characteristics of study participants (e.g. demographic, clinical, social) and information on exposures and potential confounders.<br>(b) Indicate number of participants with missing data for each variable of interest.<br>(c) Summarize follow-up time (e.g. average and total amount).		Results
Outcome data	15	Report numbers of outcome events or summary measures over time.		Results
Main results	16	(a) Give unadjusted estimates and, if applicable, confounder-adjusted estimates and their precision (e.g. 95% confidence interval). Make clear which confounders were adjusted for and why they were included.<br>(b) Report category boundaries when continuous variables were categorized.<br>(c) If relevant, consider translating estimates of relative risk into absolute risk for a meaningful time period.		Results
Other analyses	17	Report other analyses done (e.g. analyses of subgroups and interactions, and sensitivity analyses).		Results
Key results	18	Summarize key results with reference to study objectives.		Results
Limitations	19	Discuss limitations of the study, taking into account sources of potential bias or imprecision. Discuss both	(19.1) Discuss the implications of using data that were not created or collected to answer the specific research	Discussion

		direction and magnitude of any potential bias.	question(s). Include discussion of misclassification bias, unmeasured confounding, missing data, and changing eligibility over time, as they pertain to the study being reported.	
Interpretation	20	Give a cautious overall interpretation of results considering objectives, limitations, multiplicity of analyses, results from similar studies, and other relevant evidence.		Discussion
Generalizability	21	Discuss the generalizability (external validity) of the study results.		Discussion
**Other information**				
Funding	22	Give the source of funding and the role of the funders for the present study and, if applicable, for the original study on which the present article is based.		
Accessibility of protocol, raw data, and programming code		N/A	(22.1) Authors should provide information on how to access any supplemental information such as the study protocol, raw data, or programming code.	Acknowledgmen t

Supplementary Table 2. Key administrative data definitions

Variable	Database	Details	Codes
Serum creatinine	Ontario Laboratory Information System (OLIS)		OBSERVATIONCODE= 14682-9
Albumin-to-creatinine ratio	OLIS		OBSERVATIONCODE= 32294-1, 14959-1, 30000-4
Hyperkalemia (using serum potassium laboratory measurements)	OLIS<br><br>Canadian Institute for Health Information (CIHI) Discharge Abstract Database (DAD)<br>CIHI National Ambulatory Care Reporting System (NACRS)	Where the test date cannot be within a hospital admission (DAD) or an emergency department visit (NACRS).<br><br>A serum potassium measurement ≥5.5 mmol/L is evidence of hyperkalemia.	OBSERVATIONCODE=2823-3

Supplemental Table 3. Risk factors for first hyperkalemia event

Risk Factor	Hazard Ratio	95% Confidence Interval	
		Lower confidence interval	Upper confidence interval
Age (per 10 years)	0.99	0.97	1.01
Sex			
Male	0.73	0.70	0.75
Income quintile			
Quintile 1 vs. 5	1.09	1.04	1.14
Quintile 2 vs. 5	1.07	1.02	1.12
Quintile 3 vs. 5	1.06	1.01	1.11
Quintile 4 vs. 5	1.03	0.99	1.08
Index year			
2009 vs. 2008	0.73	0.68	0.79
2010 vs. 2008	0.73	0.68	0.79
2011 vs. 2008	0.75	0.70	0.81
2012 vs. 2008	0.78	0.73	0.85
2013 vs. 2008	0.83	0.77	0.90
2014 vs. 2008	1.01	0.94	1.09
2015 vs. 2008	1.13	1.04	1.23
Estimated glomerular filtration rate			
60-89 vs. ≥90 mL/min/1.73m^2	1.41	1.32	1.50
30-59 vs. ≥90 mL/min/1.73m^2	4.37	4.10	4.66
15-29 vs. ≥90 mL/min/1.73m^2	13.65	12.69	14.68
Coronary artery disease with angina	1.13	1.09	1.16
Coronary artery bypass grafting	1.00	0.92	1.09
Congestive heart failure	1.38	1.33	1.44
Myocardial infarction	1.06	1.00	1.13
Stroke & transient ischemic attack	1.08	1.00	1.17
Peripheral vascular disease	1.44	1.32	1.56
Hypertension	0.95	0.92	0.99
Diabetes	1.77	1.71	1.83
Nonsteroidal anti-inflammatory drugs	1.11	1.07	1.15
Angiotensin converting enzyme inhibitors & angiotensin-receptor blockers	1.33	1.28	1.38
Beta blockers	1.13	1.09	1.17
Statins	0.99	0.96	1.02
Potassium-sparing diuretics	1.47	1.07	2.01
Aldosterone receptor antagonists	1.29	0.94	1.77
Low molecular weight heparin	1.62	1.34	1.96
Non-potassium sparing diuretics	0.86	0.83	0.88
